# Brain functional connectivity changes by low back extension pain model in low back pain patients

**DOI:** 10.1371/journal.pone.0233858

**Published:** 2020-06-01

**Authors:** Seulgi Eun, Jeungchan Lee, Eun-Mo Song, Alexandra De Rosa, Jun-Hwan Lee, Kyungmo Park

**Affiliations:** 1 Department of Biomedical Engineering, Kyung Hee University, Yongin, Republic of Korea; 2 Center for Neuroscience Imaging Research, Institute for Basic Science, Suwon, Republic of Korea; 3 Department of Radiology, Athinoula A. Martinos Center for Biomedical Imaging, Massachusetts General Hospital, Harvard Medical School, Charlestown, Massachusetts, United States of America; 4 Department of Korean Rehabilitation Medicine, College of Korean Medicine, Kyung Hee University, Seoul, Republic of Korea; 5 Department of Biology, Massachusetts Institute of Technology, Cambridge, Massachusetts, United States of America; 6 Clinical Medicine Division, Korea Institute of Oriental Medicine, Daejeon, Republic of Korea; 7 Korean Medicine Life Science, University of Science & Technology (UST), Campus of Korea Institute of Oriental Medicine, Daejeon, Republic of Korea; Hunan Normal University, CHINA

## Abstract

**Purpose:**

Low back pain (LBP) is a common ailment in most developed countries. Because most cases of LBP are known as ‘non-specific’, it has been challenging to develop experimental pain models of LBP which reproduce patients’ clinical pain. In addition, previous models have limited applicability in a steady-pain-state neuroimaging environment. Thus, this study aims to devise a low back pain model with a simple methodology to induce experimental LBP, which has similar pain properties to patients’ clinical pain, and to apply the model in a steady-pain-state neuroimaging study.

**Methods:**

Our low back extension (LBE) pain model was tested on 217 LBP patients outside the magnetic resonance imaging (MRI) scanner to determine the reproducibility of endogenous pain and the similarity to their own clinical pain (STUDY1), and applied in a steady-pain-state functional MRI study (47 LBP patients and 23 healthy controls) to determine its applicability (induced head motions and brain functional connectivity changes; STUDY2).

**Results:**

By the LBE pain model, 68.2% of the LBP patients reported increased LBP with high similarity of sensations to their own clinical pain (STUDY1), and the head motions were statistically similar to and correlated with those in resting state (STUDY2). Furthermore, the LBE model altered brain functional connectivity by decreasing the default-mode and the sensorimotor networks, and increasing the salience network, which was significantly associated with the intensity of the induced pain. Conversely, the healthy controls showed increased somatosensory network (but not of the cognitive pain processing).

**Conclusion:**

Our investigations suggest that our LBE pain model, which increased LBP with high similarity to the LBP patients’ own pain sensation and induced patient-specific brain responses with acceptable head motion, could be applied to neuroimaging studies investigating brain responses to different levels of endogenous LBP.

## Introduction

Low Back Pain (LBP), defined by pain, stiffness, or muscle tension localized between the costal margin and the inferior gluteal folds, is common in developed countries [1], and it can be induced in muscles, ligaments, fascia, facet joints, intervertebral discs, and nerve root dura [2]. Although LBP has been classified in different ways by its duration and underlying causes [3], most cases of LBP are classified as ‘non-specific’ [4]. For this reason, most studies focused on the quantitative (e.g., pain intensity) rather than the qualitative (e.g., induced sensations) aspects of the pain [5,6,7] so that it has been challenging to develop experimental pain models reproducing LBP patients’ own clinical pain.

Although there have been many attempts to devise experimental pain models of LBP, which applied mechanical [8,9], thermal [7,10], chemical [11] stimulations on the back or limbs, these methods hardly induce pain akin to the endogenous back pain [12,13]. Nevertheless, the straight leg raise (SLR) maneuver has been considered as a sensitive test to diagnose lumbar disc herniation (sensitivity = 0.91) [14,15], and previous neuroimaging researches have reported that the SLR maneuver activates brain regions of the pain processing by exacerbating LBP [7,16,17,18].

However, it is still unclear that the SLR could be an appropriate pain model for steady-pain-state functional magnetic resonance imaging (fMRI) experiment, which investigates brain activity changes by continuously induced pain for somewhat long duration (e.g., 6 minutes). The SLR maneuver in neuroimaging studies has been usually applied to induce LBP before the MRI scanning [7,16,19]. Also, with our previous experience that tied to use the SLR maneuver in the MRI, it had a risk of generating head motions [16]. Thus, previous researches on LBP patients with the SLR maneuver were limited in resting state (i.e., induced right before the scanning but not simultaneously induced during the scanning), reporting brain functional connectivity changes in the cores of the default mode network (DMN) (e.g., medial prefrontal cortex) [5,19,20,21], the sensorimotor network (SMN) (e.g., primary somatomotor and somatosensory cortex) [22], and the salience network (SLN) (e.g., insula) [7] by the exacerbation of LBP.

In summary, LBP, as a symptom, has multiple causes, and it is difficult to develop a complete experimental pain model, which induces endogenous LBP. Although the SLR maneuver could exacerbate LBP activating brain regions of the pain processing, the neuroimaging environment (i.e., steady-pain-state fMRI) limits the applicability of the SLR maneuver. Thus, development of the pain model, which continuously reproduces endogenous LBP and is applicable in a steady-pain-state fMRI environment, is still needed for more qualified researches investigating neural mechanisms of LBP.

This study aims to validate our low back pain model, which includes a simple methodology and induces similar pain sensations to clinical pain in LBP patients for somewhat long duration (i.e., 6 minutes), and to investigate brain functional connectivity changes (i.e., DMN, SMN, SLN) by the model in a steady-pain-state fMRI environment.

## Methods

### Study designs

This study was approved by the Institutional Review Board (IRB) of Kyung Hee University and Kyung Hee University Hospital at Gangdong (IRB number: KHSIRB 2016–015 and KHNMC OH-IRB-2010-013 respectively) and publicly registered (CRIS, https://cris.nih.go.kr/, registration number: KCT0002253), and was conducted in compliance with the board’s ethical standards and with the principles of the Declaration of Helsinki. All participants understood the entire experimental protocol and provided written informed consent. This study was composed of two sub-studies. In the first study (STUDY1), we implemented and validated the experimental endogenous low back extension (LBE) pain model and investigated characteristics of the model-induced pain. Then, for the second study (STUDY2), we applied the pain model to a steady-pain-state fMRI study (1) to validate its applicability on the neuroimaging environment, and (2) to investigate brain functional connectivity changes (i.e., DMN, SMN, SLN).

### STUDY1: Implementation and validation of experimental endogenous pain model

#### Participants

A total of 217 right-handed LBP patients (126 females; 42.3 ± 14.0 years old, mean ± SD; 126 acute, < 3 months from onset date; 91 chronic, ≥ 3 months from onset date) participated in this study (STUDY1), and they were recruited from three clinical centers: Kyung Hee University Hospital at Gangdong, Mokhuri Oriental Medicine Hospital, and Sejongno Medical Clinic.

Patients were excluded from the study if they (1) were less than 19 years old, (2) had severe pain other than their back (e.g., cervicalgia and headache), (3) had severe radicular pain that extended to their lower leg (e.g., calf or foot), or (4) had LBP caused by external injuries (e.g., traffic accidents). Participant eligibility was determined in the first visit through a review of the patient’s history.

Before the pain induction test, the patients’ demographic data (e.g., age and sex) and medical history (e.g., date of LBP onset and major diagnosis) were collected. Patients were asked whether they had back pain in the flexion (bending forward) or extension (bending over backward) posture. They also rated the intensity of their endogenous LBP in the supine position without any stimulus on a visual analog scale (VAS) ranging between 0 (no pain) and 10 (the maximum imaginable pain).

#### Pain induction and outcome measures

We designed the LBE pain model, which could exacerbate the LBP using a simple methodology wedging lightly thick but not stiff foam boards (height = 4 to 7 cm, depth = 10 cm, width = 40 cm) under the supine patient’s lower back. This method, which lifts the lower back using the boards, was an attempt to emulate their clinical pain they felt upon excessive spine extension.

To estimate the extension-induced pain intensity and similarity to the clinical pain, patients were asked not to consider any pain or unpleasantness induced by the pressure or stiffness of the stimulating boards, but to only focus on the evoked LBP itself and rate its intensity on the VAS. If the pain intensity was greater than that of the resting supine position, the patient’s pain was considered increased by the LBE pain model. The similarity of the induced pain to their own clinical pain in terms of sensations was reported on a 0 to 100 scale (0: completely different sensation, 100: exactly the same sensation) to investigate if the LBE pain model could also produce or represent the patients’ own clinical pain sensations (e.g., dull, numb, sharp, throbbing, or radiating pain).

### STUDY2: Brain functional connectivity changes by the LBE pain model

#### Participants

Forty-seven right-handed LBP patients (25 males, 38.4 ± 13.0 years old, 19 acute and 28 chronic) and right-handed 23 healthy controls (HC) (11 males, 59.1 ± 5.4 years old, without a history of low back pain) were included in STUDY2. There was a significant age difference between groups (Student’s T-test P < 0.001) but not in sex (*χ*^2^ = 0.18, P = 0.67), and we used the age and sex information as controlled covariates in group-level brain data analysis. The LBP patients were recruited from STUDY1 who (1) reported increased pain by the LBE pain model to guarantee that our pain model could produce enough pain for the second study, and (2) were eligible for the fMRI scanning (e.g., no claustrophobia and pacemaker, and who could stay still for fMRI experiment in supine position). Although we could not collect the pain intensity in the resting supine position during or before the scanning, we speculated that the patients’ pain might be equal to or less than the level of 4 (VAS) according to the patients’ reports in STUDY1, which described the pain level of 4 out of 10 (VAS) or less was optimal for them to stay still for five to six minutes (the conventional fMRI experiment duration) without significant head/body movement.

#### Steady-pain stimulation in MR environment

**Fig 1:**
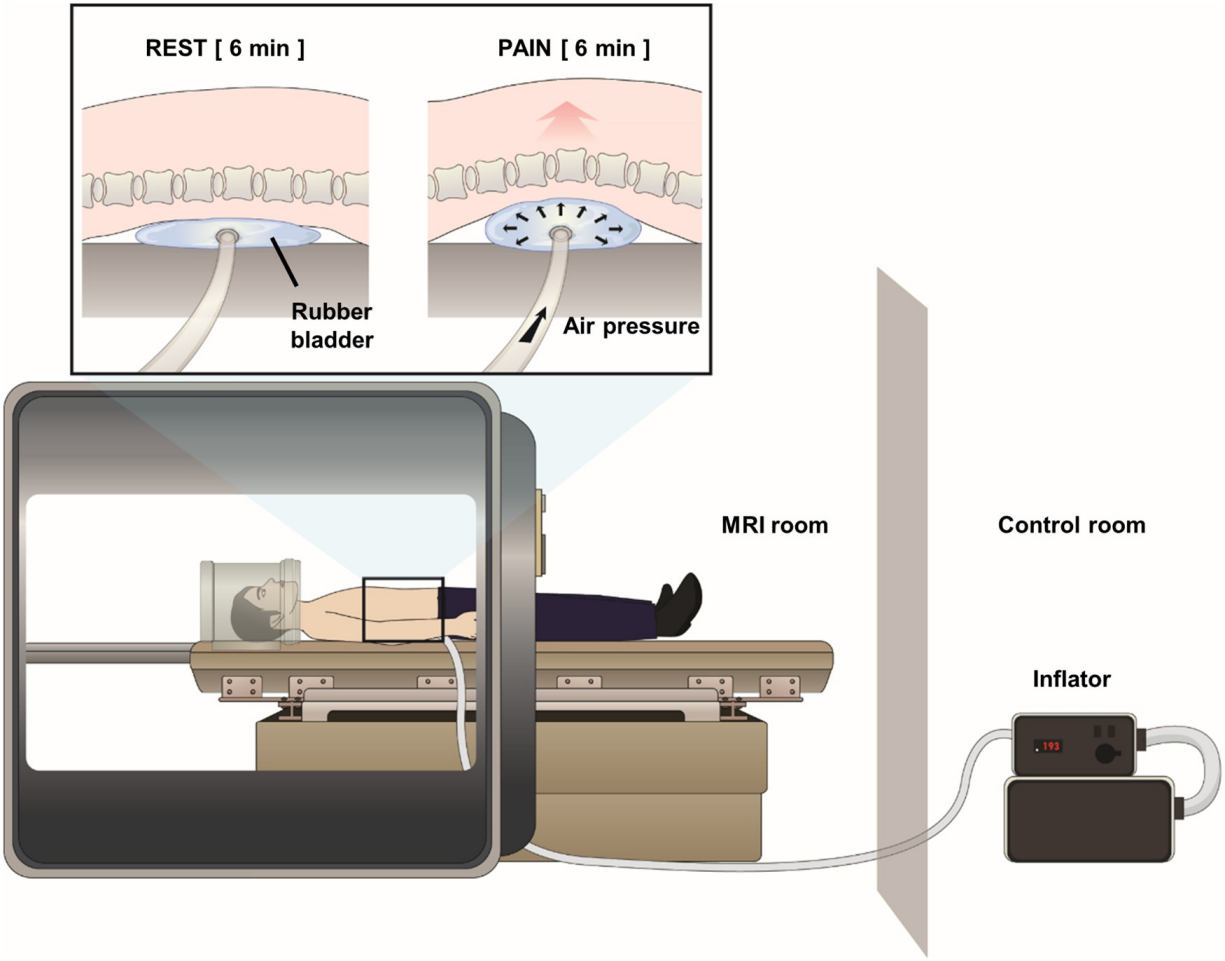
Low back extension pain model and its application in an MR environment. Air pressure was applied to the rubber bladder to allow low back extension in the supine position (in PAIN). Pressure was controlled via manual operation outside the scanner.

To test the applicability of the LBE pain model in the MR environment, a rubber bladder connected with an inflator (E20, AG101, Hokanson Inc., WA, USA) was used instead of the foam boards to apply varying levels of pressure to the lower back; by inflating the bladder under the most painful lower back area in the individual patient (under the center of lower back in HC), the extension angle of the lower back was increased to exacerbate endogenous pain (Fig 1). The rubber bladder, which was connected to the inflator through a rubber tube (through a waveguide), made this pain model applicable in such a high magnetic field environment, and the air pressure applied to the bladder could be precisely controlled according to the patient’s response (i.e., pain rating) using this method.

The amount of the applied air pressure was also individualized (target pain ratings = 4 out of 10 VAS), and the air pressure was gradually applied and removed before and after the steady-pain run to reduce any possible harm to the subjects. A fixed amount of air pressure (106 mmHg, lift up about 4 cm in a subject 183 cm tall and weighing 78 kg) was applied in the HC group based on the mean air pressure of the LBP patients (105.7 ± 64.5 mmHg). All participants remained in a supine position with their lower back lifted by the pressure under their back for a six-minute fMRI scan (see next section for more details).

#### fMRI scanning and brain data processing

The fMRI scanning was performed using a 3.0 Tesla MRI system (Achieva, Philips, Netherlands) at Kyung Hee University Hospital at Gangdong for the LBP patients and another 3.0 Tesla MRI system (Magnetom Trio, Simens, Germany) at the Brain Imaging Center at Korea University for the HC. Structural data were acquired using T1-weighted sequence (MPRAGE, TR/TE = 9,886/4.59 ms, flip angle = 8°, FOV = 256×256 mm^2^, matrix size = 256×256, slice thickness = 1 mm, 192 slices). Two steady-state fMRI runs (i.e., REST and PAIN) were collected using a T2*-weighted blood oxygen level-dependent (BOLD) echo-planar imaging (EPI) sequence in the LBP patients (TR/TE = 2,000/35 ms, flip angle = 90°, FOV = 230×230 mm^2^, matrix size = 80×80, slice thickness = 3.54 mm, 34 axial interleaved slices without gap, total scan length = 6 minutes) and the HC (TR/TE = 2,000/30 ms, flip angle = 90°, FOV = 240×240 mm^2^, matrix size = 64×64, slice thickness = 4 mm, 36 axial interleaved slices without gap, total scan length = 6 minutes). Furthermore, electrocardiogram and respiratory signals were simultaneously recorded using a conventional data acquisition system (PowerLab 16/30, ADInstruments, Australia) [23]. Subjects rested in a supine position for six minutes without any external stimulation (REST) and were stimulated with the LBE pain model for another six minutes (PAIN).

The acquired fMRI data were analyzed with the conventional analysis packages including FSL (FMRIB’s Software Library, fsl.fmrib.xo.ac.uk) [24], AFNI (Analysis of Functional NeuroImages, afni.nimh.nih.gov/afni) [25], and FreeSurfer (surfer.nmr.mgh.harvard.edu) [26].

After the fMRI data were corrected for any physiological noise (RETROICOR, AFNI) [27] and head motion (MCFLIRT, FSL) [28], the motion parameters (mean and maximum displacement and rotation) were calculated from the amount of head motion [29]. The conservative criteria for the unacceptable head motion were ≥1mm (displacement) and ≥1° (rotation) [30,31]. The data which satisfied the head motion criteria were spatially resampled (2×2×2 mm^3^) and preprocessed with skull stripping (BET, FSL), spatial smoothing (Gaussian kernel, FWHM = 6 mm) (FSLMATHS, FSL) [29], motion-related independent component removal (ICA-AROMA) [32], nuisance signal regression (including signals averaged over white matter and cerebrospinal fluid, and cardiac rate and respiratory volume per unit time respectively convolved with cardiac and respiratory response functions [33,34]), and temporal filtering (high-pass cutoff frequency = 0.006 Hz) (3dBandpass, AFNI). Individual structural and functional data were aligned (BBREGISTER, FreeSurfer), and normalized to the standard Montreal Neurological Institute (MNI) space with a non-linear registration method (FNIRT, FSL). The normalized data from the REST and the PAIN were then used in a dual-regression independent component analysis (ICA) by concatenating the data and performing group ICA (MELODIC, FSL) [35,36]. The group ICs were then spatially correlated (3D Pearson’s correlation using 3ddot, AFNI) to the standard templates for the DMN, the SMN and the SLN [37]. Three different ICs, which have the highest correlation coefficient value with each template, were chosen.

### Statistical analysis

For STUDY1, the Wilcoxon signed rank test (for the LBP intensity and the LBP-induction rate) and the Mann-Whitney test (for the group comparisons) were used to analyze the behavioral data, and Pearson’s *χ*^2^ test was applied to the demographic data using the SPSS statistical analysis program (version 18.0 for Windows, PASW Statistics, Chicago, IL, USA). In STUDY2, head motion parameters (i.e., mean and maximum displacement and rotation) were compared between REST and PAIN using the Mann-Whitney test. Also, the DMN, the SMN, and the SLN were compared (1) between groups (i.e., LBP vs. HC, age and sex were controlled for covariates), and (2) between runs (i.e., REST vs. PAIN) using FMRIB’s Local Analysis of Mixed Effects (FLAME 1, cluster-corrected for multiple comparisons, Z > 2.3, *P* < 0.05). The amount of changes in LBP intensity induced by the LBE pain model was then used to investigate relationships with the changes in brain functional connectivity within the LBP patients.

## Results

### Baseline characteristics of LBP patients

In STUDY1, the median value of the clinical pain intensity in the supine position of the 217 LBP patients was 1 (IQR = 3) on the 0–10 VAS. Among those subjects, 51 patients (23.5%) reported pain only in the flexion posture, 82 patients (37.8%) only in extension, and 44 patients (20.3%) in both (Table 1). Additional information on the diagnosis of LBP was collected from 160 patients, most (40.62%) having non-specific LBP (Table 1).

**Table 1:** Baseline characteristics of patients in STUDY1.

**Pain in supine (n = 217)**		**Median (IQR)**
Average endogenous LBP in supine position (VAS)		1 (3)
**Reported pain characteristics (n = 217)**		**N (%)**
Pain only in flexion posture		51 (23.5)
Pain only in extension posture		82 (37.8)
Pain in both postures		44 (20.3)
No pain in both postures		32 (14.7)
No records (omitted)		8 (3.7)
**Major diagnosis (n = 160)**		**N (%)**
Major diagnosis	LBP, non-specified	65 (40.62)
	Sprain and strain	58 (36.25)
	HIVD	23 (14.38)
	Other diagnoses*	14 (8.75)

IQR, Interquartile range; VAS, Visual analogue scale; LBP, Low back pain; HIVD, Herniated intervertebral disc disorder.

*Other diagnoses included spinal stenosis (n = 7), degenerative disc disorder (n = 2), spondylolisthesis (n = 2), spondylosis (n = 1), dorsalgia (n = 1), and sciatica (n = 1).

### Characteristics of the LBE model-induced LBP

In addition, 148 out of the 217 LBP patients (68.2%) reported increased pain intensity due to the LBE pain model (i.e., pain-induced group; median of Δpain = 4 with IQR = 3 VAS), while the rest of the patients (n = 69, 31.8%) rated unchanged or less pain intensity compared to the resting supine position (i.e., no-pain-induced group; median of Δpain = 0 with IQR = 1 VAS) (Table 2). Interestingly, the pain-induced group reported significantly greater LBP intensity in the resting supine position compared to the no-pain-induced group (median of pain intensity in pain-induced group = 2 with IQR = 3 VAS, in no-pain-induced group = 0 with IQR = 2 VAS, P < 0.05, Table 2). Additionally, the LBE pain model exacerbated LBP in 74.6% of the patients (94 of 126) who had pain in extension posture, while 70.5% of the patients with pain in flexion posture (67 of 95) reported exacerbated pain. The sensitivity and specificity of the LBE pain model were calculated excluding the “No records” patients, and were higher in extension posture (0.75 and 0.40, respectively) than in flexion posture (0.71 and 0.32, respectively) (Table 2).

**Table 2:** Comparison of baseline characteristics between pain-induced and no-pain-induced groups, and pain induction rates in flexion and extension postures (STUDY1).

**Total (n = 217)**	**Pain-induced (n = 148)**	**No-pain-induced (n = 69)**	***P*-value** ^a^
LBP intensity in supine (VAS)	2 (3)	0 (2)	<0.05
LBP intensity change by LBE pain model (ΔVAS)	4 (3)	0 (1)	<0.0001
**Pain characteristics**	**Pain-induced (%)**	**Sensitivity**	**Specificity**
Pain in flexion posture (n = 95)	70.5	0.71	0.32
Pain in extension posture (n = 126)	74.6	0.75	0.40

Data are shown as median (IQR).

^a^Mann-Whitney test between pain-induced and no-pain-induced groups.

**Fig 2:**
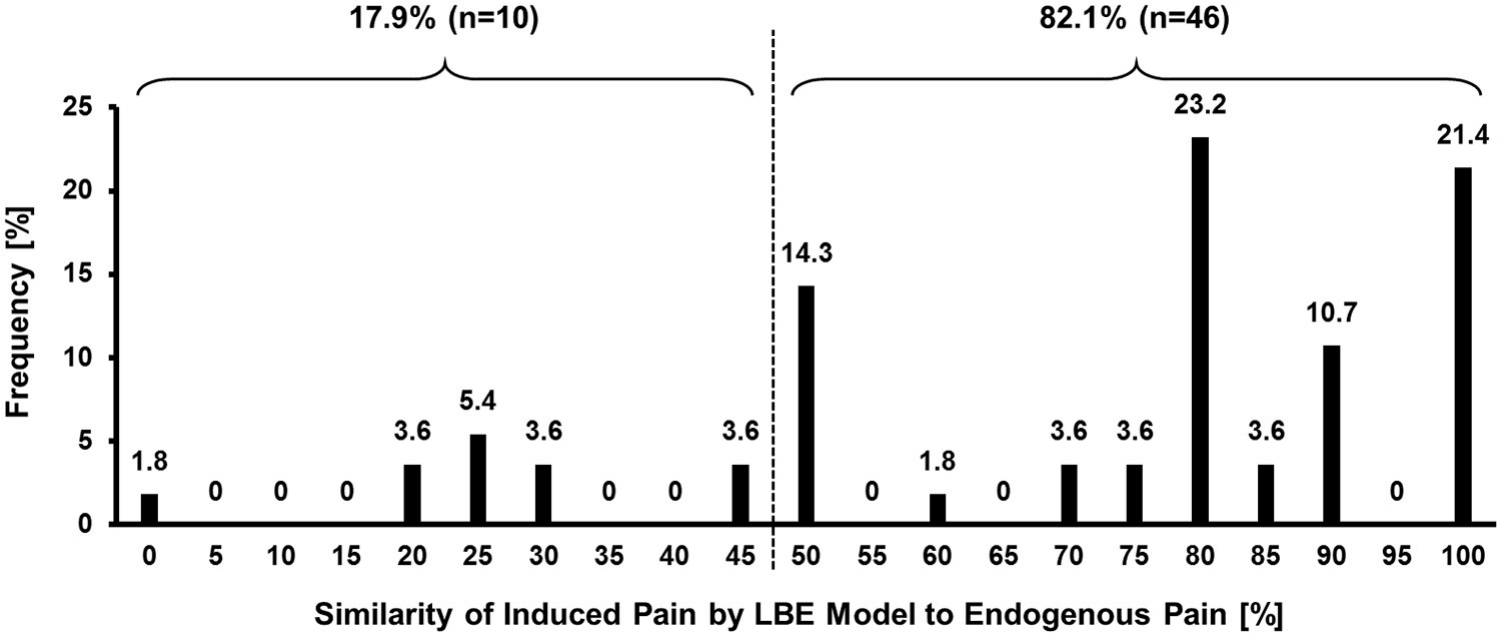
Histogram of the similarity of induced pain to clinical pain. Fifty-six subjects indicated similarity of the induced pain to their own clinical pain, and the average similarity was 69.02 ± 26.17%. Forty-six respondents (82.1%) said their induced pain was more than 50% similar to their own pain. The rest of the respondents (n = 10, 17.9%) stated that their experimental pain was less than 50% similar to their own pain.

Among the 217 LBP patients, only 56 patients reported about the similarity between the pain induced by the LBE pain model and their own clinical pain. The similarity was rated relatively high (70.8 ± 26.5%, n = 56), and 46 patients (82.1%) reported high similarity (≥ 50%) while the other 10 (17.9%) reported low similarity (< 50%) (Fig 2).

Likewise, the LBP patients in STUDY2 (n = 47) reported their pain intensity induced by the LBE pain model in the PAIN (median = 5 with IQR = 1.625 VAS) with around 60% similarity to patients’ own clinical pain (59.7 ± 23.9%). On the other hand, the LBE model did not induce any pain in the HC.

Additionally, although we do not discuss in this article, we found that there was no significant difference in the characteristics of induced pain (i.e., pain intensity, Mann-Whitney test P > 0.3; similarity, Mann-Whitney test P > 0.9; pain induction rate, *χ*^2^ test P > 0.3) but significant in age (acute = 39.4 ± 11.7 years old, chronic = 46.3 ± 15.8 years old, Mann-Whitney test P < 0.005) and duration from onset (acute = 0.05 ± 0.06 years, chronic = 4.03 ± 5.71 years, Mann-Whitney test P < 0.0000001) between acute and chronic LBP patients (STUDY1). Also, although there was no significant difference in pain similarity (acute = 60.0 ± 24.9%, chronic = 59.5 ± 23.6%, Mann-Whitney test P > 0.95) and intensity of applied pressure between acute and chronic LBP patients (acute = 104.5 ± 64.4 mmHg, chronic = 106.6 ± 65.8 mmHg, Mann-Whitney test P > 0.95), the chronic LBP patients reported significantly higher pain intensity by the LBE pain model (acute = 4.72 ± 1.02, chronic = 5.36 ± 0.89, Mann-Whitney test P < 0.05) (STUDY2).

### Head motion induced by the LBE pain model

In STUDY2, the head motion parameters (i.e., displacement and rotation, which are critical quality factors in neuroimaging data) of the REST and the PAIN were calculated to estimate the possible effect of the LBE pain model on the fMRI data quality.

**Fig 3:**
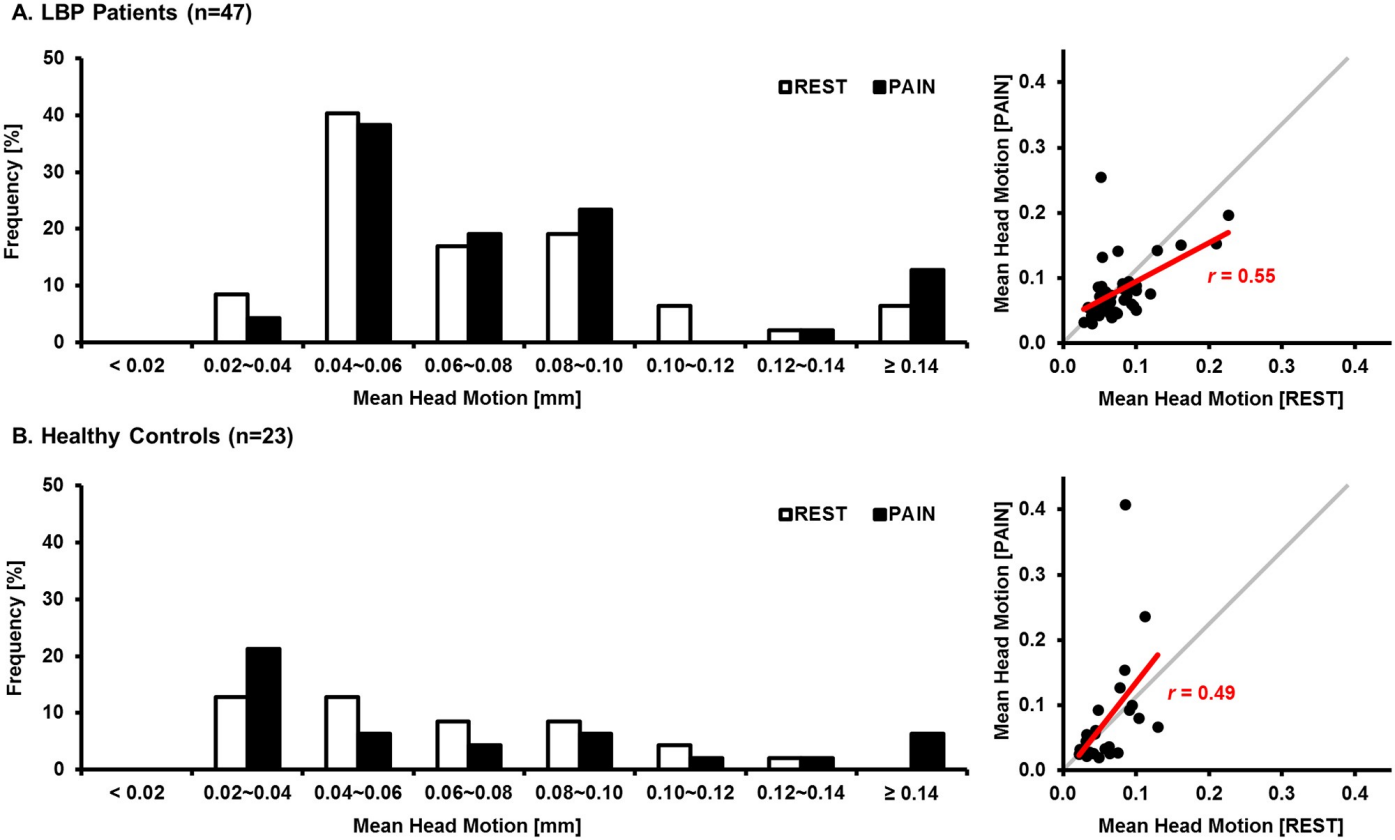
Comparison of the head motion between the REST and the PAIN in the LBP patients and the HC. Histogram of the mean head motion of the LBP patients (A, left) and HC (B, left). The white bars indicate head motion in the REST, while the black bars indicate head motion in the PAIN. The scatterplots compare the mean head motion between the REST and the PAIN in the LBP patients (A, right) and HC (B, right). In both groups, the head motion parameters in the REST and the PAIN were positively correlated (*r* = 0.55 and *r* = 0.49 respectively).

There were no significant differences in mean and maximum head motion between REST and PAIN both in LBP patients and HC (Table 3). Furthermore, we found that each parameter of REST and PAIN was positively correlated (LBP: *r* = 0.55, HC: *r* = 0.49) showing that head motion is rather an individual trait than an effect of pain stimuli (Fig 3 and Table 3).

**Table 3:** Head motion parameters of REST and PAIN in the LBP and HC groups.

Groups	Parameters	REST	PAIN	REST vs. PAIN	
				P-value^a^	Correlation^b^
**LBP patients (n = 47)**	Displacement [mm]				
	Average	0.06 (0.04)	0.07 (0.03)	0.56	0.55
	Maximum	0.30 (0.18)	0.26 (0.22)	0.47	0.56
	Rotation [°]				
	Average	0.05 (0.02)	0.05 (0.04)	0.85	0.65
	Maximum	0.21 (0.20)	0.24 (0.30)	0.61	0.55
**Healthy controls (n = 23)**	Displacement [mm]				
	Average	0.06 (0.05)	0.06 (0.07)	0.73	0.49
	Maximum	0.24 (0.19)	0.20 (0.44)	0.73	0.35
	Rotation [°]				
	Average	0.04 (0.03)	0.05 (0.05)	0.55	0.51
	Maximum	0.21 (0.18)	0.23 (0.24)	0.93	0.51

Data are shown as median (IQR).

^a^Mann-Whitney test,

^b^Pearson’s correlation coefficient between the REST and the PAIN.

Thus, although we excluded 10 subjects (7 LBP and 3 HC) whose head motion exceeded acceptable criteria (< 1 mm and < 1°) more than once during REST or PAIN in the brain functional connectivity analysis, it was not related to LBE pain model-induced motion but to qualify our results.

### Functional connectivity changes by the LBE pain model

**Fig 4:**
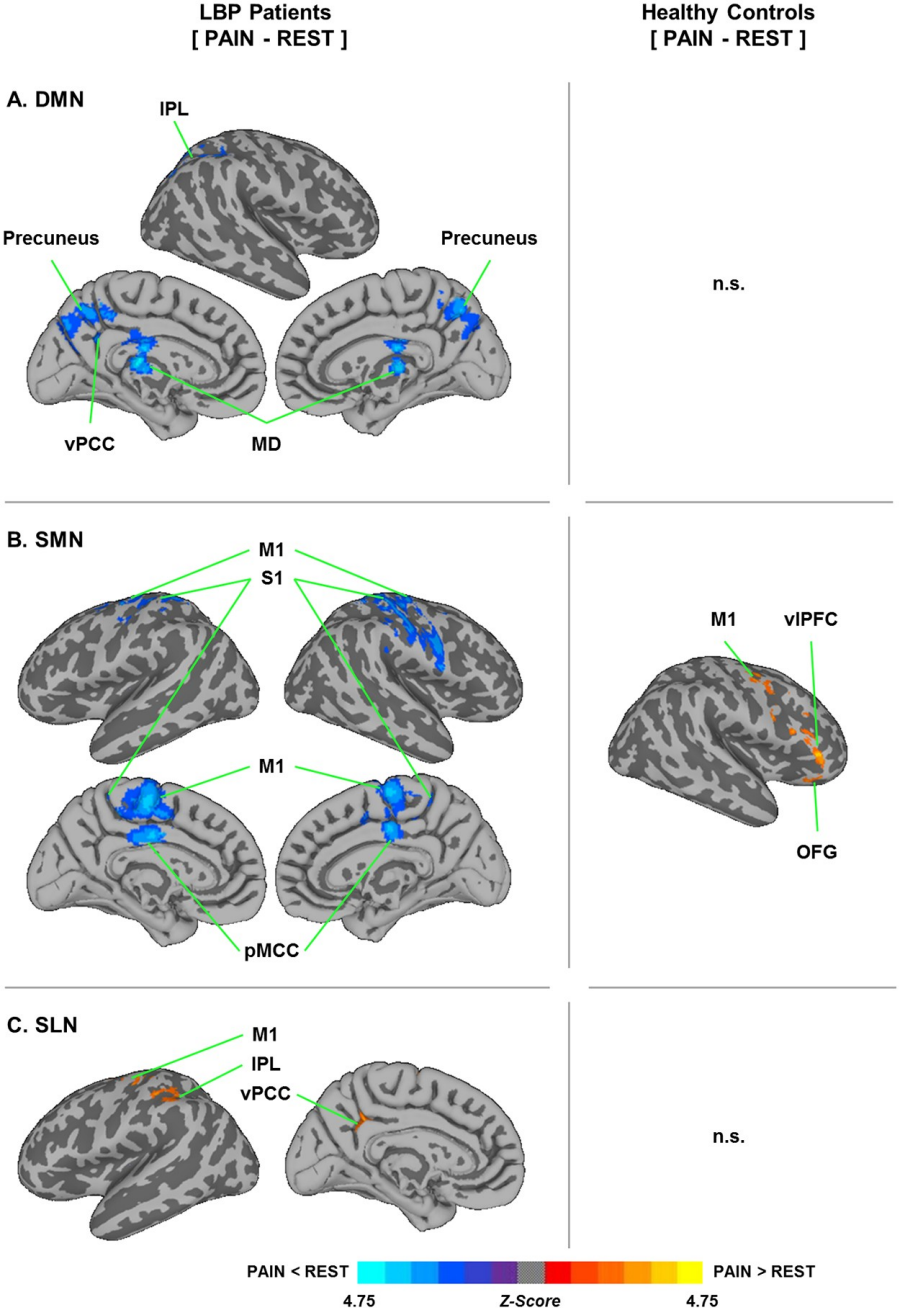
Functional connectivity changes in the PAIN compared to the REST (PAIN-REST) in the LBP patients and the HC (cluster-corrected for multiple comparisons, Z > 2.3 and P < 0.05). (A) The LBP patients showed decreased DMN connectivity of the right inferior parietal lobe (IPL), medial dorsal nucleus (MD) of the thalamus, precuneus and left ventral posterior cingulate cortex (vPCC), while there was no significant (n.s.) DMN connectivity change in the HC. (B) The LBP patients showed decreased SMN connectivity of the primary somatomotor cortex (M1) and primary somatosensory cortex (S1) in both hemispheres, and the posterior midcingulate cortex (pMCC), while the HC showed significant increases in the SMN connectivity of the right M1, right ventrolateral prefrontal cortex (vlPFC) and right orbitofrontal gyrus (OFG). (C) The LBP patients showed increased SLN connectivity of the left vPCC, left M1 and left IPL, but there were no significant changes in the HC.

In STUDY2, the LBP patients showed decreased DMN connectivity (in the right inferior parietal lobe, IPL; precuneus; ventral posterior cingulate cortex, vPCC; and medial dorsal nucleus of the thalamus, MD; Fig 4A) and SMN connectivity (in the primary motor cortex, M1; primary somatosensory cortex, S1; and posterior midcingulate cortex, pMCC; Fig 4B) in the PAIN compared to the REST, while the SLN connectivity (in the left M1, left IPL, and left vPCC) increased (Fig 4C). On the other hand, the HC showed increased SMN connectivity in the right M1, right ventrolateral prefrontal cortex (vlPFC), and right orbitofrontal gyrus (OFG) (Fig 4B).

**Fig 5:**
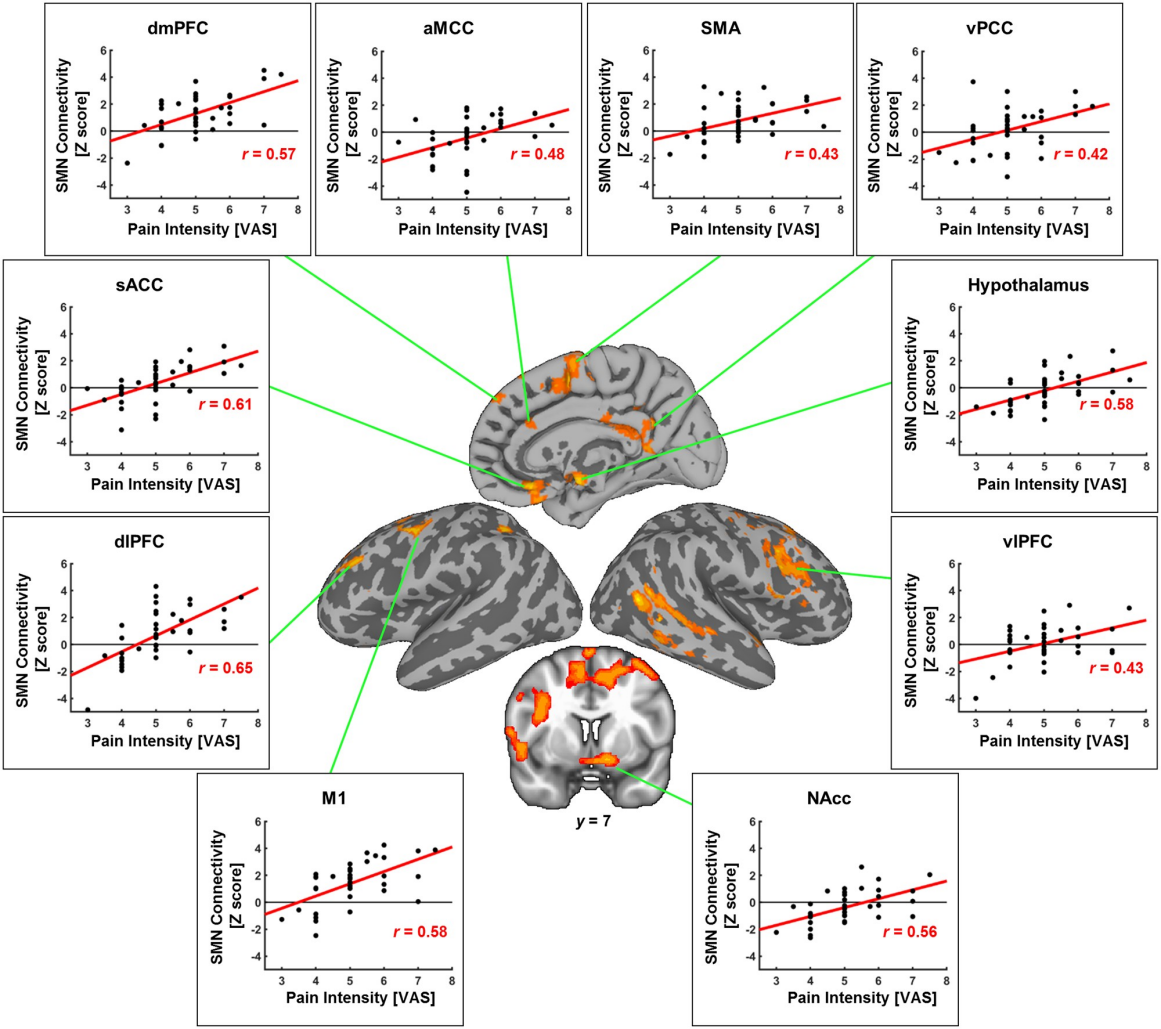
Analysis of the correlation between the LBP patients’ SMN connectivity and subjective pain intensities (VAS) in the PAIN. The SMN connectivity of the right vlPFC, right supplementary motor area (SMA), right anterior midcingulate cortex (aMCC), right vPCC, left dorsolateral prefrontal cortex (dlPFC), left M1, left nucleus accumbens (NAcc), hypothalamus, subgenual anterior cingulate cortex (sACC), and dorsomedial prefrontal cortex (dmPFC) were positively correlated to the subjective VAS (the more the pain induced by the LBE model, the more the increase in the connectivity between the SMN and these regions).

Furthermore, the increased LBP intensity induced by the LBE pain model in the LBP patients was positively correlated with the changes in the SMN connectivity (in the right vlPFC; right supplementary motor area, SMA; right anterior midcingulate cortex, aMCC; right vPCC; left dorsolateral prefrontal cortex, dlPFC; left M1; left nucleus accumbens, NAcc; hypothalamus; subgenual anterior cingulate cortex, sACC; and dorsomedial prefrontal cortex, dmPFC; Fig 5).

## Discussion

We proposed an experimental LBE pain model to modulate endogenous back pain intensity that is also applicable in a steady-pain-state fMRI experiment. Using this model, we found significantly increased clinical pain intensity with high similarity scores to the patients’ own back pain sensations and supportive neuroscientific results from the brain functional connectivity analysis.

### Exacerbated clinical back pain by the LBE pain model

In this study, we tried to exacerbate LBP by applying semi-rigid stiff boards (STUDY1) and inflating rubber bladder (STUDY2) to the participants’ lower back in a supine position. In this way, the lower back was lifted, and this produced extension-induced LBP by the excessive spine extension. Contrary to the flexion-induced LBP, which is commonly caused by vertebral fracture or prolapsed intervertebral discs, the extension-induced LBP is related to facet joint arthropathy, spinal stenosis, and other unstructured disorders [4,38]. As an unstructured and functional cause of extension-induced pain, tightness of flexor muscles (e.g., iliopsoas, rectus femoris, iliofemoral ligament, and thoracolumbar fascia) could limit low back, and the lack of muscle extensibility, lumbar curve configuration, and oxygen consumption could make these muscles shorter and limit their extension, causing extension-induced LBP [39,40].

Our proposed LBE pain model exacerbated LBP in 68.2% of the LBP patients including 74.6% of the patients who had pain in the extension posture and 70.5% of the patients who had pain in the flexion posture (Table 2). In addition, the LBE pain model showed high sensitivity of the pain induction rate both in the patients with extension-induced pain and with flexion-induced pain (0.75 and 0.71, respectively). That is, although the LBE pain model seemed to lift up the most painful area of the patient’s lower back and produce extension-induced LBP by narrowing the longitudinal angle of the spine, stretching abdomen and leg muscles and exerting pressure on the facet joints [4,41], it also aggravated flexion-induced LBP. Thus, it is speculated that the LBE pain model could be applied to LBP patients presenting, in the clinical setting, with both extension- and flexion-induced LBP.

In STUDY1, the LBE pain model increased LBP (especially in patients with high LBP in supine position, Table 2) with highly similar to their own clinical pain (similarity = 70.8 ± 26.5%). Specifically, more than 80% of patients in the pain-induced group reported high (more than 50%) similarity to their own clinical pain (Fig 2).

Thus, we speculate that the proposed LBE model, as a complementary and alternative pain model to the existing LBP models, could be applied for pain testing in clinical settings, as well as a model for pain researches.

### Applicability of the LBE pain model in the steady-pain-state neuroimaging environment

In previous steady-pain-state fMRI studies of LBP, the patient’s endogenous pain was measured by recording spontaneous pain intensity during resting-state fMRI runs, and the pain intensity fluctuation was used in the analysis [16,42]. However, during the no pain-state, the pain intensity could not be specifically controlled enough throughout the scan [42]. Also, the SLR maneuver, which has been considered as an effective maneuver to exacerbate LBP, is still needed to be validated its applicability in the steady-pain-state neuroimaging environment. Thus, in this study, we proposed an experimental LBE pain model using either non-magnetic boards (in STUDY1) or an inflating rubber bladder (in STUDY2). Importantly, this model has two advantages in the steady-pain-state neuroimaging studies: (1) the stimulation apparatus is made of non-magnetic materials, and (2) it can continuously induce LBP for comparatively long duration with acceptable range of the head motion while patients are in the supine position, which together render the model applicable to the steady-pain-state neuroimaging environment.

One of the most important factors to validate the applicability of the LBE pain model is amount of the head motion by the model. The head motion is mostly produced by changes in the head position, swallowing, and jaw clenching, and often causes motion artifact problems in neuroimaging studies [29]. The motion artifacts are problematic, as the fMRI analysis assumes that each data point (voxel) represents the activity of a fixed brain region. If the subject’s head moves during the fMRI scanning, the data are acquired from other brain regions so that data loss (usually the top of subject’s brain) or localization and estimation errors (in statistical analysis) can happen even after the head motion correction [43]. Stimulus- or task-correlated motion causes an even worse situation, because it is impossible to separate the influence of the motion from that of the stimulation or task. Thus, in this study, we could minimize the stimulus-correlated head motion and only take accounts for the pain-correlated head motion by applying the steady pain (i.e., PAIN) stimuli, rather than the on-off (blocked) pain stimulus, to the fMRI experiment.

In a recent study, the average head motion of 1,000 subjects (0.051 ± 0.004 mm) during resting-state fMRI scanning was reported [29], and it was in a range to our results (Table 3). According to recently published criteria [30,31], the movements more than 1mm in the shift and 1° in the rotation are unacceptable for the fMRI analysis. Most importantly, we found a significant correlation in the head motion parameters (e.g., displacement and rotation) between the REST and the PAIN (Fig 3 and Table 3). This implies that the head motion during the fMRI scanning was affected by an individual trait related to the control of voluntary movements, and is in the same line with previous studies, which reported that the head motion is more strongly related to the individual than to the task or the stimuli and is subject-specific to a certain degree [29,31]. Thus, we speculated that the LBE pain model might not significantly contribute to the observed head motion, which might be influenced by the innate trait of motion-control/suppression. Furthermore, we expected that the LBE pain model might be used to produce and modulate clinical pain levels in various neuroimaging studies.

### Pain-specific neural responses to the LBE pain model in LBP patients

Additionally, we found that the proposed LBE pain model triggered different brain responses (i.e., functional connectivity) in the LBP patients and the HC. In the LBP patients, the LBE pain model induced neural alternations in the cognitive processes, including increases in the attentional processes (decreased DMN connectivity to the IPL, vPCC, and precuneus) [20,44,45]. The decreases in the somatosensory processing regarding the external environmental and psychomotor contents (decreased SMN connectivity to M1, S1, and pMCC) [45] and increased salience processing for the body orienting in response to salient pain stimuli (increased SLN connectivity to M1, IPL and vPCC) [46,47,48] were also noted (Fig 4). In addition, we found significant associations between the model-induced pain intensity and the functional connectivity of the sensorimotor processing brain network (psychomotor areas: M1, SMA, and MCC; and cognitive pain processing areas: dlPFC, dmPFC, vlPFC, sACC, hypothalamus, vPCC, and NAcc) (Fig 5) [48,49,50,51,52,53,54]. On the other hand, the HC showed increased involvement of the somatosensory processing network to the brain regions for the cognitive focusing on psychomotor contents and external sensory stimuli (e.g., M1, vlPFC, and OFG) [45,55,56], but not in the cognitive pain processing area (Fig 4). Taken together, these results suggest that the LBE pain model induced patient-specific brain responses in the pain processing brain regions, which might be a putative brain feature specific for LBP.

### Study limitations

The limitations to this study should be noted. First, the working mechanisms of the LBE model seem multifactorial in terms of a large range of the stimulating targets. Even though the LBE pain model, which was designed as an extension-type model, showed a high pain-inducing rate with relatively high degree of the similarity in the patients who had extension-related pain, it is still unclear which target factors (e.g., pressed facet joints, tension of abdominal muscles, or localized pressure) of the model were critical in producing the result. Second, in some patients, the experimentally induced pain sensations were not similar to their endogenous pain, even though their pain was increased by the model. Third, this study was performed in a multicenter research trial (recruited LBP patients from more than 2 centers in STUDT1, and different MRI scanners for the LBP patients and the HC in STUDY2). As a result, some information on the diagnosis and the degree of the similarity were omitted (STUDY1), and the data quality check is needed for direct comparison between the LBP patients and the HC (STUDY2) because the type of the scanners cannot be used as a covariate in fMRI data analysis, which could limit the interpretation of the results.

## Conclusion

In conclusion, we devised a LBE pain model with a simple methodology and it produced and increased LBP with high similarity to the LBP patients’ own pain sensations. The LBE pain model was also applicable in the fMRI environment inducing only an acceptable range of head motion and produced patient-specific brain mechanisms. Our investigations suggest that our LBE pain model could be applied to neuroimaging studies investigating brain responses to different levels of endogenous LBP.

## Supporting information

S1 Dataset (ZIP)
